# Influence of Different Extraction Methods on the Changes in Bioactive Compound Composition and Antioxidant Properties of Solid-State Fermented Coffee Husk Extracts

**DOI:** 10.1155/2023/6698056

**Published:** 2023-09-21

**Authors:** Rossaporn Jiamjariyatam, Pakkapong Phucharoenrak, Siritron Samosorn, Kulvadee Dolsophon, Wanlapa Lorliam, Sukhumaporn Krajangsang, Prapakorn Tantayotai

**Affiliations:** ^1^Department of Chemistry, Faculty of Science, Srinakharinwirot University, 114 Sukhumvit 23, Bangkok 10110, Thailand; ^2^Institute of Nutrition, Mahidol University, Nakhon Pathom 73170, Thailand; ^3^Department of Microbiology, Faculty of Science, Srinakharinwirot University, 114 Sukhumvit 23, Bangkok 10110, Thailand

## Abstract

In brewing coffee, a huge amount of food waste is generated; that waste, coffee husks in particular, should be comprehensively exploited. They offer a rich source of bioactive compounds such as caffeine, chlorogenic acid, and trigonelline. The aim of this study was to investigate the effects of extraction methods on the bioactive compounds and antioxidant activity of such waste. Coffee husks in this study were fermented with *S. cerevisiae* based on a solid-state fermentation technique. The study method included ethanolic or water extraction with varied controllable factors, i.e., temperature (60, 100°C) and extraction technique. Bioactive contents were investigated with the Folin–Ciocalteu assay and ^1^H-NMR spectroscopy. The antioxidant activity was investigated with DPPH and FRAP assays. Results show that yields were the highest in the extract of fermented coffee husks at 100°C. The highest levels of bioactive contents (total trigonelline content at 3.59% and antioxidant activity at 23.35% (DPPH) and 25.9% (FRAP)) were found in the ethanolic extract of fermented coffee husks at 60°C. The bioactive content and bioactivity, including antioxidant activity, depended on different raw materials, preparation methods, and extraction conditions. This study illustrates the potential for using food waste such as coffee husks as a sustainable source of bioactive compounds or bioactive extracts.

## 1. Introduction

Coffee is one of the most popular nonalcoholic beverages in the world. It has a unique aroma, flavor, and taste, which result from a complex combination of more than 1,000 chemical components [1], although the predominant bioactive compounds in coffee are caffeine, caffeic acid, chlorogenic acids, trigonelline, diterpenes, and melanoidins, many of which have been scientifically proven to promote or improve health. In fact, coffee itself has many reported health benefits. It is recognized as a health drink when consumed in appropriate amounts [2]. In addition to the expected health benefits of drinking coffee, coffee is a crop that is important to the global economy. It is cultivated in more than 70 countries worldwide; the global coffee market in 2022 was valued at 503.86 billion USD and is expected to reach 797.12 billion USD by 2030 [3]. In 2021, the estimated global consumption of coffee beans was approximately 10.25 million metric tons, and demand is generally increasing every year [4]. However, coffee production also produces an incredible amount of biological waste since the most commonly consumed part of coffee is the bean; the remainder ends up as food waste that results from processing, accounting for approximately 8.45 million metric tons or around 45.2% of the total weight of coffee fruit. The waste product includes the skin, pulp, mucilage, parchment, and silver bean [5]. Coffee husks offer great potential to decrease the industry's carbon footprint while providing value to consumers via upcycled food products. Some of these have a history of being consumed as traditional foods or beverages (pulp), while some parts (e.g., husk and silver bean) have been studied to assess their safety and benefits as novel foods. Previous studies have found that waste products from coffee processing offer a source of nutrients, dietary fibers, and bioactive compounds that are not inferior to the coffee beans themselves [6, 7]. However, it has been reported that the disposal of coffee waste products can have severe ecotoxicological effects. Coffee grounds can reduce the levels of oxygen in the water and earth, resulting in the death of local fauna due to lack of oxygen or an increasingly acidic environment. In addition, this waste can emit 28.6 million tons of carbon dioxide per year and results in the release of methane into the atmosphere, exacerbating the climate crisis [8, 9].

The primary solid waste product from coffee processing is coffee husk, which contains both nutrients and nonnutrients, including carbohydrates (35–85%), proteins (5–11%), fat (0.3–5.5%), dietary fiber (24–43%), minerals (K, Ca, Mg, Zn, Cu, and Fe), polyphenol, flavonoid, and other bioactive compounds [10, 11]. The prominent bioactive compounds found in coffee husks are caffeine, chlorogenic acid, and trigonelline [12]. Chlorogenic acid is an important bioactive polyphenol that is responsible for the bitter and astringent taste of coffee. It is one of the most abundant phenolic acids and is found in green coffee beans rather than roasted coffee beans [13]. Trigonelline is a plant alkaloid formed by a methylation reaction of niacin (vitamin B3). Previous review articles have concluded that chlorogenic acid and trigonelline have pharmacological activities (e.g., antioxidant, anti-inflammatory, antimutagenic, antitumor, antidiabetic, antiobesity, antihypertension, hepatoprotective, nephroprotective, cardioprotective, and neuroprotective) and thus play a role in reducing the risk of various diseases [14]. Solid-state fermentation is a process that involves the cultivation of microorganisms on a solid matrix (raw materials) in conditions that are without water or nearly free water. Previous studies have reported that the solid-state fermentation process can increase the content and bioactivity of bioactive compounds. It has been found that solid-state fermentation of coffee grounds can increase the total flavonoid, total phenolic, chlorogenic acid, quinic acid, and caffeic acid content [15–17]. The process may offer the potential to develop functional ingredients for health benefits in the future. However, some bioactive compounds, including chlorogenic acid and trigonelline, are unstable and easily degraded, especially when exposed to heat or light [18]. In addition, there are alternative extraction methods that use sustainable source materials or environment-friendly solvents (i.e., ethanol and water) and deliver an extract rich in bioactive compounds; these methods seem promising, and it will be necessary to study their usefulness for reducing environmental problems and promoting sustainable development [19]. Therefore, the present study aimed to investigate the effect of optimal and simple green extraction of bioactive compounds from fermented coffee husks [20]. In addition, utilizing coffee husk, which is a food waste product from coffee processing, is one way to reduce the amount of food waste and decrease its negative impact on the environment [20]. This complies with sustainable development goals in goal 12: responsible consumption and production and goal 13: climate action [21].

## 2. Materials and Methods

### 2.1. Chemicals and Raw Materials

Ethanol, sulfuric acid, and phosphoric acid, all of which were of analytical grade from Honeywell (Seelze, Germany), were used in the process. Sodium metabisulfite, used to stabilize the chlorogenic acids during the assays, was of analytical grade and was purchased from Sigma Aldrich (Steinheim, Germany). Chlorogenic acid was purchased from Biopurify (Chengdu, China). Trigonelline, sodium 3-(trimethylsilyl)-[2,2,3,3-d4]-1-propionate (TSP), deuterium oxide, and other chemicals used were purchased from Merck Millipore (Burlington, Massachusetts, USA). Coffee husks from the wet depulping and demucilaging process of coffee beans (*Coffea arabica*) were cleaned until water was clear. Raw coffee husk was split into lots of 3 kg and kept in a freezer at −20 degrees Celsius prior to experiments; batches of 1 kg of coffee husk were thawed at room temperature when needed.

### 2.2. Preparation of Fermented Coffee Husk

Fermented coffee husk was used in this study. It was prepared by a solid-state fermentation method adapted from a previous study [22]. Arabica coffee husk was thawed from freezing at −20 degrees Celsius to room temperature and then blended with a Thermomix TM31 multifunction disintegrator (Vorwerk, Wuppertal, Germany) at maximum speed for 1 minute. Ground coffee husk was fermented with 10 ml activated yeast (1 g of *S. cerevisiae*) in a sealed bag at room temperature for 8 hours in the dark, with a solid-to-solvent ratio of 0.1 g/ml. After the fermentation period, 0.5% sodium metabisulfite (Na_2_S_2_O_5_) was added to inactivate yeast, and the mixture was kept in a freezer at −20 degrees Celsius until ready for use in the study.

### 2.3. Experimental Design

Five different extraction methods were designed and tested in this study: (I) extraction from fermented coffee husk by distilled water at 100 degrees Celsius for 20 minutes, (II) extraction from fermented coffee husk by distilled water at 60 degrees Celsius for 20 minutes, (III) extraction from fresh coffee husk by distilled water at 60 degrees Celsius for 20 minutes, (IV) extraction from fermented coffee husk by ethanol, using a reflux technique at 60 degrees Celsius for 20 minutes, and (V) extraction from fermented coffee husk soaked with ethanol inside a closed container at room temperature for 6 days. All extraction methods were performed with a solid-to-solvent ratio of 0.5 g/ml. After extraction periods, the liquid was filtered from the coffee husk. The extract was then dried in a hot air oven at 60 degrees Celsius until the samples were completely dry (for ethanolic extracts, ethanol was evaporated in a rotary evaporator before drying). The weights of all samples were recorded, and the percentage of yield was calculated. Each sample was stored in an airtight container in a dark and dry place at 25 degrees Celsius until it was ready for analysis.

### 2.4. Determination of Total Phenolic Contents

Total phenolic content was analyzed using the Folin–Ciocalteu reagent adapted from a previous study [23]. The extracts were mixed with saturated sodium bicarbonate and 10% Folin–Ciocalteu reagent (v/v). This solution was then incubated in the dark at room temperature for 30 minutes. After that, the absorbance was measured at a wavelength of 765 nm by using a DU-8800D spectrophotometer (QTECH, Russia). The total phenolic content was presented as gallic acid equivalents (GAE) in milligrams per gram of sample extract (mg·GAE/g).

### 2.5. Determination of Antioxidant Activities

Antioxidant assays were performed using ferric ion reducing antioxidant power (FRAP) and 2,2-diphenyl-1-picrylhydrazyl (DPPH) radical scavenging by a colorimetric assay adapted from a previous study [24, 25]. For the FRAP assay, the extracts were mixed with FRAP reagent that was freshly prepared by mixing 300 mM acetate buffer (pH 3.6), 20 mM FeCl_3_·6H_2_O solution, and 10 mM 2,4,6-tris (2-pyridyl)-s-triazine (TPTZ in 40 mM HCl) at the ratio of 10 : 1 : 1. The mixture was incubated for 4 minutes at room temperature before measuring the absorbance at the wavelength of 593 nm by using a DU-8800D spectrophotometer (QTECH, Russia). The electron donating capacity of ferric ion (Fe^3+^) was presented as a ferrous ion (Fe^2+^) equivalent in millimolars per gram of sample extract or percent inhibition by comparison with the standard curve of ferrous sulfate (FeSO_4_). The DPPH assay mixture consisted of the extracts and DPPH solution (250 *μ*g/ml of DPPH in ethanol). It was placed into a 96 well-plate and incubated in the dark at room temperature for 30 minutes. After that, the absorbance was measured at the wavelength of 517 nm by using an RT-2100C microplate reader and SoftMax Pro Software analysis software (Meditech, Australia). The free radical scavenging capacity was presented as a percent inhibition calculated from the absorbance loss of DPPH radicals. Ascorbic acid was used as the standard for this assay, and the extract concentrations were in the range of the ascorbic acid standard curve.

### 2.6. NMR-Analysis of Trigonelline Content

Six trigonelline standard concentrations were obtained by two serial dilutions in the range of 0.06–2.00 mg/ml. 10 mg of coffee husk extract (one sample from each extraction method) was dissolved with 4 ml of warm water to 2.5 mg/ml and then filtered through a 0.22 *µ*m nylon syringe filter. To 800 *µ*L of each trigonelline concentration or coffee husk extract filtrate was added 100 *µ*L of phosphate buffer (pH 6) and 100 *µ*L of 0.05% TSP in deuterium oxide (D_2_O). All samples were placed into a quartz NMR tube and analyzed by ^1^H NMR in triplicate, using TSP as a reference standard. The ^1^H NMR experiments were performed on a Bruker Advance NEO 500 MHz (Billerica, Massachusetts, USA) with noesygppr1d pulse program. Spectra were recorded into 64 K complex points over a 29.4040 ppm spectral width and averaged over 64 scans after 4 dummy scans, NMR Data Processing by Bruker Topspin 4.1.3.

### 2.7. Statistical Analysis

Statistical analyses were performed using PASW Statistics 18 (formerly SPSS Statistics) (Chicago, Illinois, USA) or GraphPad prism V.9 (San Diego, California, USA). Data were expressed as means ± SD of at least 3 replications (*n* ≥ 3). One-way analysis of variance (ANOVA) with the Tukey post hoc test was performed to compare the mean of each experimental condition. A *p* value <0.05 indicated a statistically significant difference.

## 3. Results and Discussion

### 3.1. Percentage of Extraction Yields

Extraction yields from each extraction method were presented in percentages that were calculated from the ratio of extract weights to coffee husk weight (shown in Table 1). The extract yield was found to be the highest in the sample that used water extraction of fermented coffee husk at 100 degrees Celsius (method I: 5.1%) and the lowest in the sample that macerated fermented coffee husk in ethanol for 6 days (method V: 0.8%). It was found that, at the same temperature, the extraction yield by the water-solvent method was higher than the ethanol-solvent method. However, in the same extraction solvent, increasing the temperature resulted in a higher extraction yield. At least one previous study had reported that the type of extraction solvent and temperature were important factors that affected the efficiency of plant extraction [20]. In fact, the solvent has a great influence on the yield of the extract, and it determines the type of components that can be extracted and dissolved in the extract. This is because of differences in the polarity of the extraction solvents, which have an effect on the solubility of the components in the raw material [26]. Generally, heating is expected to increase the extraction efficiency because the solubility of compounds should increase with the rise in temperature [27]. In addition, this study also demonstrated that under the same extraction conditions, fermented coffee husk yielded less than unfermented coffee husk. Furthermore, when using the same extraction method (reflux technique with ethanol), the extraction yield in this study (1.1%) was lower than the extraction yield in previous studies (4.8–27.58%) [28, 29], which may have resulted from the use of different raw materials for extraction (fermented or fresh coffee husk). Consistently, previous studies have found that the type of raw material was one of the factors that influenced the extraction yield as did other extraction factors, i.e., extraction time, solid-to-solvent ratio, and pH value of the solvent [20, 30] It would be valuable to carry out further studies aimed at understanding the relationships between the extraction factors in order to increase the extraction efficiency.

### 3.2. Total Phenolic Contents

All coffee husk extracts exhibited total phenolic contents in the range of 0.0002–0.0082 mg·GAE/g dry weight of extract (shown in Table 1). The extracts obtained using ethanol extraction tended to exhibit the highest total phenolic contents. This method provided total phenolic contents up to 20-fold higher than water extraction. According to the results, water extraction seemed to be less effective for the extraction of phenolics. This result is consistent with a previous study that reported a tendency for total phenolic and total flavonoid contents to increase with an increase in ethanol concentration in the extraction solvent [31]. This may be a result of the polarity of the extraction solvent having an effect on the solubility of compounds. The solubility of phenol compounds in the extraction solvent is a very influential factor on the total phenolic contents of extracts. Previous studies reported that the total phenolic content of coffee husk extracts was in the range of 0.0118–4.5459 mg·GAE/g, depending on different raw materials and extraction conditions [32, 33]. Thus, the extraction efficiency is dependent on the selection of the optimal solvent, temperatures, and mechanical agitation for use in the extraction process [34]. However, it has been reported that the use of mixed solvents, e.g., water-ethanol can extract more bioactive compounds than pure solvents [20, 35, 36]. However, reducing the solvent volume through reuse can be difficult when using mixed solvents, especially in small and medium industries. Ethanol can form a binary azeotrope with water in mixed solvents [37]. As a result, the water cannot be completely removed using simple distillation techniques. Therefore, it is difficult to recycle ethanol when using mixed solvents. To obtain pure ethanol for reuse, the ternary system technique required a substantial quantity of energy [37, 38]. Previous studies reported that sequential extractions (from nonpolar to polar solvents) can improve the extraction efficiency and uncovering of widely phytochemical compounds and bioactivity [39, 40]. However, the use of a wide variety of extraction solvents required proper waste management. This involved costs, logistics, laws, practices, storage capacity, safety considerations, and technology available in the production area, when scaled up from laboratory to industry scale [41]. Consequently, we designed the study using only pure solvents (water or ethanol) so that it can be scaled up to a small industry scale, which is the limitation of this work.

### 3.3. Antioxidant Activities

Oxidation is a very complicated mechanism, so a single method cannot be relied on to completely evaluate the antioxidant properties. In this study, the antioxidant properties of coffee husk extracts were determined by DPPH and FRAP assays, and the results are shown in Table 2. The antioxidant properties from both assays were found to be the highest in the sample that used water extraction of fermented coffee husk at 100 degrees Celsius (method III), followed by the sample that used ethanol-reflux extraction of fermented coffee husk at 60 degrees Celsius (method IV). The lowest levels of antioxidant properties were found in the sample that used water extraction of fermented coffee husk at 60 degrees Celsius (method II). In general, bioactive compounds are present in the bound form with the matrix; thus, the high temperature was able to break down interactions between the bioactive compounds and the matrix [42]. As a result, antioxidant properties were increased in samples that were extracted under higher temperature. However, the rise in temperature may decrease the bioactivity of the extract because; at the high-temperature condition, some bioactive compounds could suffer degradation or a loss of function [20]. From the results, fresh coffee husk extract was found to have higher antioxidant properties than fermented coffee husk extract. This may be the result of yeast metabolism that can decompose or change some components of the raw material during the fermentation process. Consistently, previous studies have identified the effects of fermentation on various components (including phenolics). Specifically, fermentation can reduce the contents or change the structure of bioactive compounds such as catechin content or lead to the conversion of sinapine to sinapic acid [43–45]. However, the results with respect to the antioxidant properties were not the same as those of total phenolic content, which indicates that the coffee husk extracts also contain other compound groups that have antioxidant properties in addition to phenolic acid. Further studies on the phytochemical profile of the coffee husk extracts are needed. In addition, the antioxidant property assay that was carried out in this study is based on the electron transfer (ET) mechanism only. Therefore, further studies with assays based on the hydrogen atom transfer mechanism should be performed to more concisely evaluate antioxidant properties.

### 3.4. NMR Analysis of Chlorogenic Acid and Trigonelline

**Figure 1:**
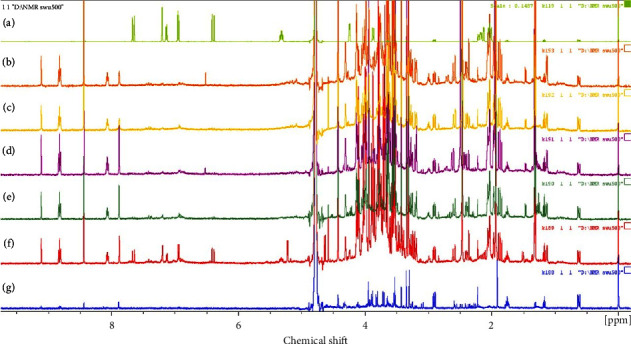
^1^H NMR fingerprints of (a) chlorogenic acid standard; (b) fermented coffee husk was extracted with water at 60°C; (c) fermented coffee husk was extracted with water at 100°C; (d) fermented coffee husk was extracted with ethanol at 60°C; (e) fermented coffee husk was soaked with ethanol; (f) fresh coffee husk was extracted with water at 60°C; (g) fresh ground coffee husk.

The ^1^H NMR spectra proposed as fingerprints are shown in Figure 1. The chlorogenic acid peak in the chemical shift region from 5.3 to 7.7 ppm stands out. The results indicate that a chlorogenic acid peak can be identified in the extraction from fresh coffee husks, while it is rarely detected in extracts from fermented coffee husks. This contradicts a previous study that reported that solid-state fermentation may increase chlorogenic acid content by up to 400% [22], perhaps due to a result of free water content generated in the process of thawing frozen coffee husk from −20 degree Celsius to room temperature. Water is one of the necessary factors for yeast activity. Its presence may have resulted in increased fermentation and reproduction of yeast in the solid-state fermentation processes, which would be consistent with a previous study that found that chlorogenic acid content in coffee beans decreased after refermentation processes [46]. In addition, the water content in the fermentation system was an important factor affecting the regulation of chlorogenic acid degradation kinetics. The presence of water in the process results in the conversion of chlorogenic acid to caffeic acid through a hydrolysis reaction [47]. In the result of the ^1^H NMR analysis, a peak of trigonelline was found to stand out in the chemical shift region from 8.0 to 9.2 ppm. Trigonelline is the major alkaloid found in coffee. A previous study reported that trigonelline content was significantly decreased during the fermentation period [16]. However, with respect to the fermentation time, this study was shorter (8 hours) than that of the previous studies (24–168 hours) [16, 48]. This is one factor that may have led to different amounts of trigonelline. Thus, a quantitative investigation of trigonelline is required.

### 3.5. Total Trigonelline Content

**Figure 2:**
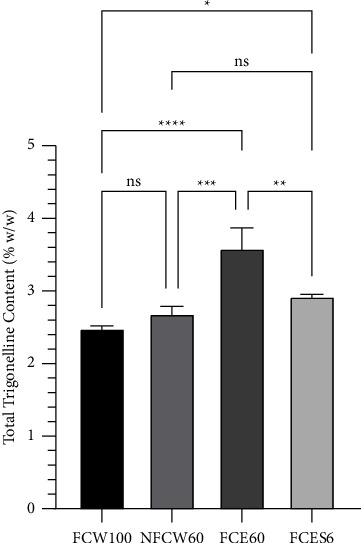
Total trigonelline content (% w/w) of water extract of fermented coffee husk at 100°C (FCW100), water extract of fresh coffee husk at 60°C (NFCW60), ethanolic extract of fermented coffee husk by the reflux technique at 60°C (FCE60), and ethanolic extract of fermented coffee husk by the maceration technique for 6 days (FCES6). The statistical differences were analyzed by using one-way ANOVA followed by the Tukey post hoc test. ns, ^*∗*^, ^*∗∗*^, ^*∗∗∗*^, and ^*∗∗∗∗*^ mean *p* > 0.05, ≤0.05, <0.01, <0.001, and <0.0001, respectively.

A quantitative assessment for the determination of trigonelline in coffee husk extract was performed on the ^1^H NMR signal of trigonelline H-2 (9.2 ppm, singlet). Calibration curves were employed to calculate the concentrations of trigonelline in the extracts, which were found to be in the range of 2.46–3.87% (w/w) (shown in Figure 2), and were the highest in the sample that used ethanol-reflux extraction of fermented coffee husk at 60 degrees Celsius (method IV). This indicates that coffee husks are a good source of trigonelline when compared to coffee beans (around 1% w/w) [49]. In fact, trigonelline is highly soluble in water and hot ethanol [50]. However, from the results, trigonelline was found to be higher in the ethanolic extract than in the aquatic extract. This may be the result of water contained in the coffee husk sample. Consistently, previous studies have reported that the presence of water in the extraction solvent causes the plant cells to swell, allowing easier diffusion of solvent into the plant cells. In addition, the polarity of the solvent can be adjusted to be highly suitable for the dissolution of trigonelline [51, 52]. In the same extraction solvent, we found that an increase in extraction temperature did not have any effect on the trigonelline content due to the fact that trigonelline is a heat-stable alkaloid that does not undergo thermal degradation during the extraction process. However, it may not be possible to extract all the trigonelline from the matrix at 100°C for 20 min [53]. It is possible that the trigonelline content of the extract in this study was lower than the actual trigonelline content in the coffee husk. In this study, we did not find a significant difference between fresh and fermented coffee husks.

## 4. Conclusion

This study demonstrates that coffee husk obtained as food waste from coffee processing offers a potential renewable plant source for bioactive extracts. Coffee husk extracts exhibit antioxidant properties based on an electron transfer mechanism and contain high levels of trigonelline content compared to coffee that is generally consumed. The study found that the optimal extraction method relied on ethanol-reflux extraction of fermented coffee husk at 60 degrees Celsius, which provided an extraction yield of 1.1%, total phenolic content of 0.008 mg·GAE/g, and total trigonelline content of 3.59%. The antioxidant activity of this extract as measured by the DPPH assay and FRAP assay was measured at 23.35% and 25.79%, respectively. This study did not find a difference between fresh and fermented coffee husk extracts. However, it was found that the condition of sample preparation and extraction factors (including temperature, type of solvent, extraction technique, and extraction time) had a significant effect on antioxidant properties and bioactive compound content. In particular, the water content is an important factor in the chlorogenic acid content which control the hydrolysis of caffeic acid. It would be worthwhile to conduct further studies to understand the relationships between the extraction factors in order to increase the efficiency of the process. The findings of this study are the effect of the extraction methods on the antioxidant activity and the content of bioactive compounds, especially trigonelline. This may lead to the development of the extraction method with high health benefits for coffee husk extracts as antioxidants, anti-inflammatory, antiaging agents, etc. However, although coffee husk extract has drawn interest as a functional ingredient for potential health benefits, further studies should be conducted to assess its potential for further utilization.

## Figures and Tables

**Table 1:** Extraction yields and total phenolic content of the lyophilized samples of coffee husks by each extraction method.

Extraction conditions	Extraction yield (% w/w)	TPCs (mg·GAE/g)
Method (I): Water extraction of fermented coffee husk at 100°C	5.1	0.0005^b^ ± 0.0003
Method (II): Water extraction of fermented coffee husk at 60°C	2.5	0.0004^c^ ± 0.0002
Method (III): Water extraction of fresh coffee husk at 60°C	4.2	0.0005^b^ ± 0.0001
Method (IV): Ethanol-reflux extraction of fermented coffee husk at 60°C	1.1	0.0080^a^ ± 0.0001
Method (V): Maceration of fermented coffee husk in ethanol for 6 days	0.8	0.0080^a^ ± 0.0002
Ground fresh coffee husks	—	0.0004^c^ ± 0.0002

It shows mean ± SD (*n* ≥ 3). Different superscript letters, *p* ≤ 0.05; same letter (*p* > 0.05), obtained from ANOVA with the Tukey post hoc test.

**Table 2:** Antioxidant activities detected by 2,2-diphenyl-1-picrylhydrazyl (DPPH) radical scavenging and ferric ion reducing antioxidant power (FRAP) of coffee husk extracts.

Extraction conditions	DPPH (% inhibition)	FRAP (% inhibition)
Method (I): Water extraction of fermented coffee husk at 100°C	20.31^bcd^ ± 1.63	21.46^bcd^ ± 1.53
Method (II): Water extraction of fermented coffee husk at 60°C	17.17^cd^ ± 0.86	16.56^cd^ ± 2.56
Method (III): Water extraction of fresh coffee husk at 60°C	32.00^a^ ± 5.27	35.73^a^ ± 1.25
Method (IV): Ethanol-reflux extraction of fermented coffee husk at 60°C	25.35^ab^ ± 0.61	25.79^ab^ ± 2.61
Method (V): Maceration of fermented coffee husk in ethanol for 6 days	23.35^bc^ ± 3.76	26.01^bc^ ± 1.45
Ground coffee husk	15.34^d^ ± 3.38	12.95^d^ ± 1.22

It shows the mean ± SD (*n* ≥ 3). Different superscript letters, *p* ≤ 0.05; same letter (*p* > 0.05), obtained from ANOVA with the Tukey post hoc test.

## Data Availability

The data that support the findings of this study are available from the corresponding authors upon reasonable request.

## References

[1] Jeszka-Skowron M., Zgoła-Grześkowiak A., Grześkowiak T. (2015). Analytical methods applied for the characterization and the determination of bioactive compounds in coffee. *European Food Research and Technology*.

[2] Bułdak R. J., Hejmo T., Osowski M. (2018). The impact of coffee and its selected bioactive compounds on the development and progression of colorectal cancer in vivo and in vitro. *Molecules*.

[3] ResearchandMarkets (2023). *Coffee Market Research Report by Coffee Bean (Arabica, Excelsa, and Liberica), Product Type, Distribution, Region-Cumulative Impact of COVID-19, Russia Ukraine Conflict, and High Inflation-Global Forecast 2023-2030*.

[4] Ico (2022). *Monthly Coffee Market Report-January 2023*.

[5] Aristizábal-Marulanda V., Chacón-Perez Y., Cardona Alzate C. A., Galanakis C. M. (2017). Chapter 3- the biorefinery concept for the industrial valorization of coffee processing by-products. *Handbook of Coffee Processing By-Products*.

[6] Esquivel P., Jiménez V. M. (2012). Functional properties of coffee and coffee by-products. *Food Research International*.

[7] Klingel T., Kremer J. I., Gottstein V., Rajcic de Rezende T., Schwarz S., Lachenmeier D. W. (2020). A review of coffee by-products including leaf, flower, cherry, husk, silver skin, and spent grounds as novel foods within the European union. *Foods*.

[8] Navia P D. P., Velasco M R. D. J., Hoyos C J. L. (2011). Production and evaluation of ethanol from coffee processing by-products. *Vitae*.

[9] Lauberts M., Mierina I., Pals M., Latheef M. A. A., Shishkin A. (2023). Spent coffee grounds valorization in biorefinery context to obtain valuable products using different extraction approaches and solvents. *Plants*.

[10] Bondesson E. (2015). *A Nutritional Analysis On The By-Product Coffee Husk And Its Potential Utilization In Food Production*.

[11] Alhogbi B. G. (2017). Potential of coffee husk biomass waste for the adsorption of Pb(II) ion from aqueous solutions. *Sustainable Chemistry and Pharmacy*.

[12] Cangussu L. B., Melo J. C., Franca A. S., Oliveira L. S. (2021). Chemical characterization of coffee husks, a by-product of coffea arabica production. *Foods*.

[13] Narita Y., Inouye K. (2015). Chlorogenic acids from coffee: coffee in health and disease prevention. *Elsevier*.

[14] Naveed M., Hejazi V., Abbas M. (2018). Chlorogenic acid (CGA): a pharmacological review and call for further research. *Biomedicine & Pharmacotherapy*.

[15] Rochín-Medina J. J., Ramírez K., Rangel-Peraza J. G., Bustos-Terrones Y. A. (2018). Increase of content and bioactivity of total phenolic compounds from spent coffee grounds through solid state fermentation by Bacillus clausii. *Journal of Food Science and Technology*.

[16] Myo H., Nantarat N., Khat-Udomkiri N. (2021). Changes in bioactive compounds of coffee pulp through fermentation-based biotransformation using Lactobacillus plantarum TISTR 543 and its antioxidant activities. *Fermentation*.

[17] Arancibia-Díaz A., Astudillo-Castro C., Altamirano C. (2023). Development of solid-state fermentation process of spent coffee grounds for the differentiated obtaining of chlorogenic, quinic, and caffeic acids. *Journal of the Science of Food and Agriculture*.

[18] Heo J., Adhikari K., Choi K. S., Lee J. (2020). Analysis of caffeine, chlorogenic acid, trigonelline, and volatile compounds in cold brew coffee using high-performance liquid chromatography and solid-phase microextraction-gas chromatography-mass spectrometry. *Foods*.

[19] Chemat F., Vian M. A., Cravotto G. (2012). Green extraction of natural products: concept and principles. *International Journal of Molecular Sciences*.

[20] Phucharoenrak P., Muangnoi C., Trachootham D. (2022). A green extraction method to achieve the highest yield of limonin and hesperidin from lime peel powder (*Citrus aurantifolia*). *Molecules*.

[21] UnitedNations (2022). *The Sustainable Development Goals Report 2022*.

[22] Santos da Silveira J., Durand N., Lacour S. (2019). Solid-state fermentation as a sustainable method for coffee pulp treatment and production of an extract rich in chlorogenic acids. *Food and Bioproducts Processing*.

[23] Kujala T. S., Loponen J. M., Klika K. D., Pihlaja K. (2000). Phenolics and betacyanins in red beetroot (*Beta vulgaris*) root: distribution and effect of cold storage on the content of total phenolics and three individual compounds. *Journal of Agricultural and Food Chemistry*.

[24] Benzie I. F. F., Strain J. J. (1996). The ferric reducing ability of plasma (FRAP) as a measure of “antioxidant power”: the FRAP assay. *Analytical Biochemistry*.

[25] Fukumoto L. R., Mazza G. (2000). Assessing antioxidant and prooxidant activities of phenolic compounds. *Journal of Agricultural and Food Chemistry*.

[26] Truong D.-H., Nguyen D. H., Ta N. T. A., Bui A. V., Do T. H., Nguyen H. C. (2019). Evaluation of the use of different solvents for phytochemical constituents, antioxidants, and *in vitro* anti-inflammatory activities of Severinia buxifolia. *Journal of Food Quality*.

[27] Ma Y., Ye X., Hao Y., Xu G., Xu G., Liu D. (2008). Ultrasound-assisted extraction of hesperidin from Penggan (Citrus reticulata) peel. *Ultrasonics Sonochemistry*.

[28] Andrade K. S., Gonçalvez R. T., Maraschin M., Ribeiro-do-Valle R. M., Martínez J., Ferreira S. R. (2012). Supercritical fluid extraction from spent coffee grounds and coffee husks: antioxidant activity and effect of operational variables on extract composition. *Talanta*.

[29] Raharjani S. A., Jessica A., Angela Justina K., Agus C., Abduh M. Y. (2021). Effect of extraction conditions on yield and bioactive compounds of coffee pulp extract. *Biological and Natural Resources Engineering Journal*.

[30] Babbar N., Oberoi H. S., Uppal D. S., Patil R. T. (2011). Total phenolic content and antioxidant capacity of extracts obtained from six important fruit residues. *Food Research International*.

[31] Sun C., Wu Z., Wang Z., Zhang H. (2015). Effect of ethanol/water solvents on phenolic profiles and antioxidant properties of Beijing propolis extracts. *Evidence-Based Complementary and Alternative Medicine*.

[32] Neves J. V. G. D., Borges M. V., Silva D. D. M. (2019). Total phenolic content and primary antioxidant capacity of aqueous extracts of coffee husk: chemical evaluation and beverage development. *Food Science and Technology*.

[33] Saewan N., Jimtaisong D. A., Vichit W. (2020). Optimization of phenolic extraction from coffee by−product using response surface methodology and their antioxidant activities. *Food and Applied Bioscience Journal*.

[34] Kim D.-O., Lee C. Y. (2002). Extraction and isolation of polyphenolics. *Current Protocols in Food Analytical Chemistry*.

[35] Li B. B., Smith B., Hossain M. M. (2006). Extraction of phenolics from citrus peels: I. Solvent extraction method. *Separation and Purification Technology*.

[36] Inthachat W., Temviriyanukul P., On-Nom N. (2023). Optimization of phytochemical-rich citrus maxima albedo extract using response surface methodology. *Molecules*.

[37] Gordon W. A., Summary P. P. (1986). Method and apparatus for removing water from ethanol. *National Center for Biotechnology Information*.

[38] Megawati D. W., Abdullah M. S. (2017). Experimental study on the adsorptive-distillation for dehydration of ethanol-water mixture using natural and synthetic zeolites. *AIP Conference Proceedings*.

[39] Agatonovic-Kustrin S., Morton D., Mizaton H., Zakaria H. (2018). The relationship between major polyphenolic acids and stigmasterol to antioxidant activity in different extracts of Myrmecodia platytyrea. *South African Journal of Botany*.

[40] Swain A., Santhoshkannada A., Prabakaran D., Choudhir G., Puttaswamy H. (2021). Phytochemical and pharmacological exploration of Cyperus articulatus as a potential source of nutraceuticals and drug ingredients. *Indian Journal of Pharmaceutical Education and Research*.

[41] Seyler C., Capello C., Hellweg S. (2006). Waste-solvent management as an element of green chemistry: a comprehensive study on the Swiss chemical industry. *Industrial & Engineering Chemistry Research*.

[42] Jeong S.-M., Kim S.-Y., Kim D.-R. (2004). Effect of heat treatment on the antioxidant activity of extracts from citrus peels. *Journal of Agricultural and Food Chemistry*.

[43] Vlassa M., Filip M., Ţăranu I. (2022). The yeast fermentation effect on content of bioactive, nutritional and anti-nutritional factors in rapeseed meal. *Foods*.

[44] Lücke F.-K., Fritz V., Tannhäuser K., Arya A. (2019). Controlled fermentation of rapeseed presscake by Rhizopus, and its effect on some components with relevance to human nutrition. *Food Research International*.

[45] Niu Y., Jiang M., Guo M. (2015). Characterization of the factors that influence sinapine concentration in rapeseed meal during fermentation. *PLoS One*.

[46] Sinaga H. L. R., Bastian F., Syarifuddin A. (2021). Effect of decaffeination and re-fermentation on level of caffeine, chlorogenic acid and total acid in green bean robusta coffee. *IOP Conference Series: Earth and Environmental Science*.

[47] Vandeponseele A., Draye M., Piot C., Chatel G. (2020). Subcritical water and supercritical carbon dioxide: efficient and selective eco-compatible solvents for coffee and coffee by-products valorization. *Green Chemistry*.

[48] Sandhya M. V. S., Yallappa B. S., Varadaraj M. C. (2016). Inoculum of the starter consortia and interactive metabolic process in enhancing quality of cocoa bean (Theobroma cacao) fermentation. *LWT-Food Science and Technology*.

[49] Trugo L. C., Caballero B. (2003). COFFEE | analysis of coffee products. *Encyclopedia of Food Sciences and Nutrition*.

[50] Mohamadi N., Sharififar F., Pournamdari M., Ansari M. (2018). A review on biosynthesis, analytical techniques, and pharmacological activities of trigonelline as a plant alkaloid. *Journal of Dietary Supplements*.

[51] Gertenbach D. D., John Shi G. M., Le Maguer M. (2002). Solid–liquid extraction technologies for manufacturing nutraceuticals. *Functional Foods*.

[52] Hussein L. A., Abdel Ghany M. F., Yamani H. Z. (2015). Development of microwave-assisted extraction of trigonelline biomarker from trigonella foenum-graecum seeds followed by high-performance thin-layer chromatographic and high-performance liquid chromatographic analyses. *JPC-Journal of Planar Chromatography- Modern TLC*.

[53] Sasaki M., Nonoshita Y., Kajiya T. (2020). Characteristic analysis of trigonelline contained in raphanus sativus cv. Sakurajima daikon and results from the first trial examining its vasodilator properties in humans. *Nutrients*.

